# Assessing the effects of price regulation and freedom of choice on quality: evidence from the physiotherapy market

**DOI:** 10.1186/s13561-017-0158-2

**Published:** 2017-06-23

**Authors:** Piia Pekola, Ismo Linnosmaa, Hennamari Mikkola

**Affiliations:** 1 Social Insurance Institution of Finland, PL 450, 00056 Helsinki, Finland; 2 National Institute for Health and Welfare, PL 30, 00271 Helsinki, Finland

**Keywords:** Competitive bidding, Financial incentives, Physiotherapy, Quality, Regulation, Service voucher, I11, I18, L15, L51

## Abstract

In health care, many aspects of the delivery of services are subject to regulation. Often the purpose of the regulated health care system is to encourage providers to keep costs down without skimping on quality. The purpose of this paper is to analyse the effect of price regulation and free choice on quality in physiotherapy organised by the Social Insurance Institution of Finland for the disabled individuals.

We use the difference-in-differences method in our effort to isolate the effect of the regulation and for this task we have defined the regulated and non-regulated firms and their quality before and after the regulation. The variables needed in the econometric modelling were collected from several registers as well as by carrying out questionnaires on the firms.

We show that price regulation decreased quality in physiotherapy statistically significantly and the mechanism was unable to incentivise firms to invest in quality. Most likely, our results are caused by cost reduction associated with price regulation. It seems that cost reduction was carried out through quality reductions in physiotherapy instead of increasing productivity. The result is sensible because comparable quality information is not published to support patient choice in this sector.

## Background

The main purpose of the regulated health care system is to encourage providers to keep costs down without skimping on quality. Also when government agencies or insurers are purchasing health services they usually try to keep costs down without decreasing quality [1]. Yet, due to changes in the financial incentives, firms may alter their behavior regarding quality. This means that firms may have an incentive to decrease quality in order to cut the costs instead of improving productivity [2]. For previous reasons, price regulation is recommended in the literature to be linked to elements affecting competition such as free choice. In this scenario, competition will outweigh a firm’s possible incentive to seek cost reductions through quality [3].

Previously mentioned plans to regulate health care prices linked with free choice have already been piloted in Finland in rehabilitation and especially in physiotherapy services organised and financed by the Social Insurance Institution (Kela). Kela has a supplemental role in the Finnish health care sector and it is a largest single organiser of rehabilitation services in Finland. Generally, Kela uses public procurement mechanisms such as competitive bidding in its effort of organising rehabilitation services. However, during the contract period 2011-2014, fixed price service vouchers were piloted in two insurance districts in physiotherapy targeted at disabled individuals. Additionally, free choice was initiated during the same period throughout the country.

Due to price regulation and free choice (henceforth also reform), the system potentially had a huge impact on the financial incentives of firms. Firms which were located in the two insurance districts where service vouchers were piloted had regulated prices. Whilst firms located in all other districts were able to define prices in their tenders during competitive bidding. Thus the reform in physiotherapy had two opposite incentives for firms: price regulation may induce firms to cut costs by decreasing quality, but free choice may lead to increased quality due to competition.

The purpose of this paper is to analyse the effects of price regulation and free choice on quality in physiotherapy organised and financed by Kela for the disabled individuals in Finland. The study is novel - it provides evidence from rehabilitation and especially physiotherapy from which there are no previous studies. Thus, we aim to broaden the literature in this respect but it is also useful for the future purposes in Finland from which there are no previous studies whatsoever in this sector. As Finland is planning to reform its health and social care sector (and especially primary health care) by introducing fixed prices and enlarging free choice to public, private and third sector providers it is useful to have knowledge from previous reforms as well.

In the previous literature, it has been shown that changes in reimbursement influence providers’ incentives towards the intensity of care provided (i.e., quality of care) or patient selection [4]. Shen has analysed the effect of financial pressure on hospital quality [5]. The study demonstrated that the effect of financial pressure on quality might differ depending on the type of competition that dominates the market. Dafny on the other hand, analysed the responses of hospitals to changes in DRG (Diagnoses-related Group) pricing and found that hospitals responded to changed prices by upcoding more patients into groups in which prices had increased the most. However, the hospitals did not increase admissions differently for those diagnoses with the largest price increases and foremost, the regulator could not positively influence the quality produced by the hospitals [6].

Sood et al. analysed the change in the prospective payment system (PPS) for inpatient rehabilitation facilities (IRF) and its effect on marginal and average reimbursement. The results show that the new PPS led to a significant reduction in costs and length of stay, but had little or no impact on outcomes, e.g., mortality or the rate of return to residence in the community [7]. In a more recent study, Allen et al. analysed activity-based financing systems (Best Practice Tariffs) and found that this system incentivised hospitals to reduce unit costs and it may even facilitate patient choice and competition, but could also reduce quality if patient choice is unable to respond to quality [8]. In another study, California patient discharge data and hospital financial disclosure reports were analysed to explore the effects of competition under prospective payment on hospital costs for low and high-cost admissions within the 12 largest DRGs. With using the DiD method, researchers were able to show that increased competition was associated with increased costs before the price regulation but the effect decreased later, especially amongst high-cost patients. Interestingly similar effects were found on high-cost patients both above and below 65 years. The findings support the idea that a fixed price scheme created an incentive to reduce expenditures on high-cost patients [3].

We are interested in the effects of price regulation and free choice on quality in physiotherapy. We use Difference-in-Differences (DiD) regression in our effort to isolate the effect of the regulation, and for this task, we have defined the regulated and non-regulated firms (666 regulated firms and 58 non-regulated firms) and their quality before and after the reform. We have also added other firm and market structure (municipality) level variables such as potential patient capacity and the amount of patients in a municipality to the analyses as control variables. We finalise our analyses with a kernel matching.

### Theoretical background

Price regulation of health care providers e.g. hospitals is aimed to lower costs or at least to reduce the rate of hospital cost inflation without skimping on quality. The intuition behind prospective payment system (PPS), or any other fixed price system for that matter, is that providers will be incentivized to use less resource in treating patients and providers that have lower costs than the flat rate would benefit from the system compared to hospitals with higher costs. However, there are concerns that fixed prices such as PPS would induce hospitals to save on costs by cream skimming and/or reducing quality etc. [9]. Because of the tradeoff between intended and unintended outcomes, it is important to combine regulation and competition in health care because it has been shown that competition with non-regulated prices tends to increase costs and price regulation without competition has no financial incentive to increase quality [3].

The main purpose of the price regulation in physiotherapy in Finland was the implementation of service vouchers, which in the case of physiotherapy for disabled individuals, must not include out-of-pocket payments and were thus a fixed price system. Because also patient choice was initiated at the same time in physiotherapy, in addition to the primary function, the scheme had the capacity to support competition and increase efficiency as price regulation steers service providers to compete for patients on non-price dimensions such as quality.

When prices are regulated, prices do not have a strategic role and competition among providers is solved via quality to increase market share [10]. Prevailing theory regarding fixed prices strongly suggests that quality increases as more competitors enter the market - assuming that the regulated price is above marginal costs, that marginal costs are constant, that profit margin is positive, firms are profit maximisers and demand will be responsive to quality [11, 12].

Increased competition (with fixed prices) means that a higher density of firms are providing services in the market [13]. When the number of firms in the market increases, the demand of a firm becomes more elastic and, therefore, firms choose higher quality in order to attract more customers [14]. The magnitude of this increase in quality is defined by the quality and elasticity of the demand [15].

Despite the strong theoretical prediction with the general theory of competition and quality with fixed prices, the most recent literature shows that, e.g., a provider’s altruism, increasing marginal costs and imperfect information may decrease the positive effect that competition might have on quality. In the case of altruism, firms are interested in patients’ wellbeing and eventually this behavior will decrease their effort of growing profits. Brekke et al. has shown that with semi-altruistic providers there is an unambiguous relationship between increased patient choice and service quality. In this case, patient choice has two contradicting outcomes. A more quality responsive demand increases the incentives to decrease quality so that financially unprofitable patients would choose other providers. On the other hand, an altruistic provider wants to increase quality and thus patient benefit. Researchers have shown that depending on the size of the conflicting effects, competition will either decrease or increase quality [13].

The larger the profit margin, the stronger are firms’ incentives to increase quality. Thus, also the increase in the regulated prices will increase the marginal net profit from higher quality. Increasing marginal costs on the other hand, diminishes firms’ incentives to engage in quality competition and the reason behind increasing costs may be capacity constraints. If patient capacity is limited, firms must either abstain from quality competition (i.e., increasing volume) or invest in extra capacity which will be increasing marginal costs. Therefore, the profit margin (and thus the incentive to compete for patients) will also be greater if the level of the fixed price includes investment costs [12].

Finally, information affects patient’s responsiveness towards service quality. If patients start reacting to increased quality information (i.e., quality differences) intuitively this would also affect providers’ incentives towards pro-competitive direction [12]. Gravelle & Sivey (2010) have demonstrated that only if providers have similar quality and thus similar costs, increased quality information will improve quality. A similar result occurs if information is initially relatively imprecise. On the other hand, if quality differences are large between/amongst providers, cost differences also tend to be large. In this situation, with improved information, patients are making even more accurate decisions regarding providers and with fixed prices marginal revenues are also small and thus there is no incentive to increase quality in either of the hospitals [16]. Gravelle & Masiero have studied quality incentives in a regulated market with imperfect information in their theoretical study regarding general practitioners (GPs). The study shows that for any given regulated capitation fee, quality is lower and the incentive effects on quality are smaller when there is imperfect information [17].

As can be seen from previous theoretical literature, the effect of price regulation combined with quality competition potentially leads quality in two opposite directions. Price regulation may induce firms to cut costs by reducing quality while intensified competition may incentivise firms to increase quality. As information is presumed to be imperfect in our study, the quality outcome due to reform in physiotherapy is unambiguous and by using empirical data we aim to test which effect dominates in the market.

### Physiotherapy market

Public health care is universal and tax financed in Finland, but Kela has a supplementary role in the health sector as it also organises and finances health services such as rehabilitation. Kela is obliged by law to organise medical rehabilitation such as physiotherapy for disabled persons who fulfil the criteria defined in the law (Rehabilitation law 566/2005) and the institution is one of the largest organisers and financiers of rehabilitation services in Finland. In 2011, Kela had in total approximately 1,320 service providers of physiotherapy. During the same year, the annual costs of physiotherapy were approximately 50 million euros [18].

In addition to the physiotherapy services studied, there are also other ways to organise physiotherapy services in Finland. Physiotherapy may be provided as part of the public health care or by part of the occupation health care. In addition, a large part of the services are provided by private physiotherapists (firms) and patients are entitled to small subsidies from Kela. Yet, this market is not controlled by Kela and Kela is not involved in organising these services. Instead, patients using these services make the selection of providers themselves and thus the market formation is very different from the one studied. Approximately 40% of all physiotherapy firms had a contract with Kela to produce services for the disabled individuals and these services constitute about 22% of all physiotherapy provided in Finland in 2011 [19].

Kela uses public procurement mechanisms, mainly competitive bidding, when organising physiotherapy (or other rehabilitation services). Services are purchased from the private sector. In physiotherapy, during the contract period 2007-2010 all services were organised with competitive bidding, but during the contract period 2011-2014, fixed-price service vouchers were piloted in two insurance districts while 23 insurance districts continued organising competitive bidding.

A service voucher pilot, i.e. price regulation as well as the introduction of patient choice, was introduced by policy change in the procurement. Thus the fixed price system associated with patient choice was an administrative decision made by Kela. The request for registration, including information on the level of regulated prices for the pilot districts, was announced (before the pilot began) in September 2010 for the contract period 2011–2014.

Price regulation was geographically defined by Kela. The aim was to have a sufficiently competitive environment (adequate amount of supply and demand) but exclude the largest insurance districts as well as the geographically most challenging districts. During the contract period 2011–2014, a firm was able to participate in either the voucher system or in competitive bidding (depending on the insurance district they operated on) but not in both.

**Fig. 1:**
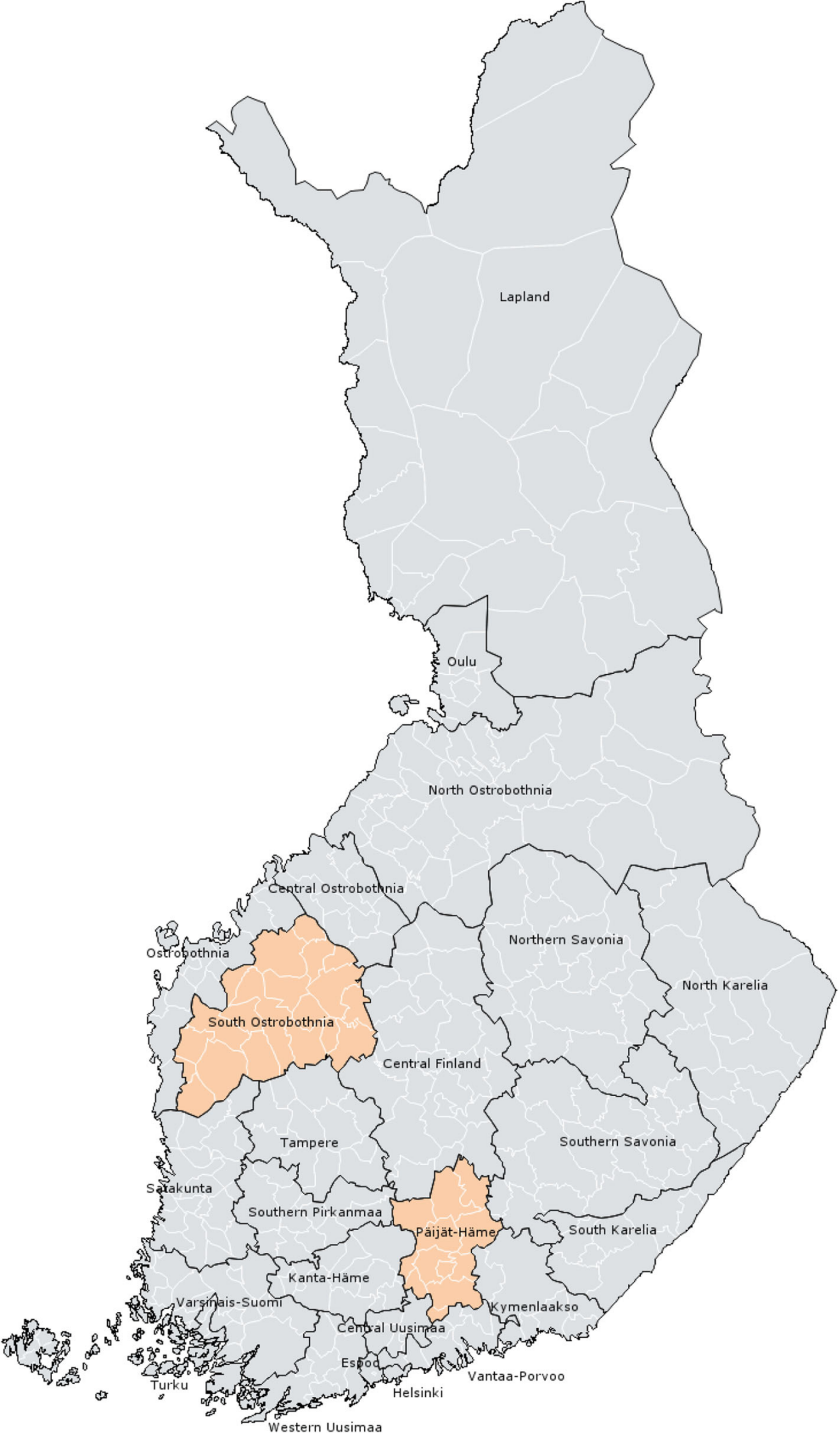
Kela insurance districts in 2011. Service voucher was piloted in two districs – Päijät-Häme and South Ostrobothnia

The chosen pilot areas had some general features: the two districts were geographically located in different parts of Finland and they could be described as medium-sized districts, which included 31 municipalities. From those 31 municipalities, Kela had service providers in 26 municipalities and there were between 1 and 22 providers in each. In 2011, the two pilot districts had 118 providers in total, which is approximately 9.5% of all firms contracted to provide services for disabled individuals. Pilot districts are presented in Fig. 1.

The procurement process is very different between competitive bidding and fixed-price service vouchers. The major difference is the pricing – the service voucher scheme had regulated prices while in competitive bidding, prices were defined by firms in their tenders. With competitive bidding, the minimum quality of the service, as well as other requirements for the service providers were defined in the request for tender. Firms set their price for a 45-min therapy session, while taking into account quality and capacity in their tenders. During the procurement process, Kela scored each firm’s price and quality (education, work experience and premises, as well as its quality, the quality of the equipment and the extent to which it conformed to Kela’s quality standard) in a predefined manner. After completing the procurement process, qualified firms were accepted to join a pool of firms. Patients then choose proper service providers based on their individual preferences.

With service vouchers on the other hand, separate registration processes were carried out in both of the two Kela insurance districts where fixed price service vouchers were operational. All service providers who accepted the regulated price and who fulfilled certain predefined minimum quality criteria^1^ were eligible to produce physiotherapy and these firms received written contracts with Kela for the contract period 2011-2014. A firm which declines to accept regulated prices and/or fails to meet minimum quality requirements will not be contracted with Kela and thus may not produce physiotherapy for the disabled individuals financed from NHI. The purpose of the registration process was to create a pool of eligible firms for both of the two insurance districts (or 26 municipalities). After completing registration, patients choose their service providers among eligible firms. The differences between the two different procurement mechanisms are defined in Table 1.

**Table 1:** The differences between competitive bidding and service vouchers as procurement mechanisms

Process	Competitive bidding	Service voucher
Price	Defined by firms in their tenders	Regulated by Kela
Minimum quality and other criteria	Controlled by Kela	Controlled by Kela
Excess quality	Scored during the procurement process	Not scored or evaluated during registration
Contracting	Completed with firms based on quality-price ratio	Completed with all firms fulfilling minimum criteria
Patient choice	Patients may choose a local provider that has a contract with Kela	Patients may choose a local provider that has a contract with Kela
Out-of-pocker payments required	No	No

During the contract periods 2007–2010 and 2011–2014 medical rehabilitation was arranged by Kela if the patient fulfilled the criteria set in the rehabilitation law. A written rehabilitation plan forms the basis for medical rehabilitation services for persons with severe disabilities. The plan is drawn up with the doctor in charge of the patient’s health care. Physiotherapy for disabled individuals is granted based on individual needs and requirements, the maximum being once a week for up to three years at a time.

In 2011 approximately 14,000 persons received physiotherapy services targeted at disabled individuals. In general, disabled individuals receiving physiotherapy services ranged from young children to adults up to 65 years of age. The median age of the patients was 43 years [18]. In 2011 there were approximately 1,200 (750 and 450) patients receiving these services in the two districts where fixed price service vouchers were piloted.

In physiotherapy studied, out-of-pocket payments are not required and money follows patients. Patient choice is effective throughout the country as patients may choose proper service providers from the pool of firms based on their individual preferences despite how the firms are contracted. Despite Kela covering (reasonable) travel costs to the patients, the distance to the provider is important because the service is targeted at the disabled individuals. Therefore, patients are presumed to make decisions among/between providers within their own municipalities. Based on the previously described operational environment, patients’ decision-making is based on distance to the provider and quality of the service. However, Kela provides only a smidgen of information about firms to patients and information is considered imperfect in this study.

Based on recent study, over 95% of the disabled individuals (adults) receiving physiotherapy studied appreciate the right to choose a provider based on their individual preferences. Approximately 45% of the respondents pointed out that they are able choose a provider individually. Thus, despite of disabilities, this patient group must be considered as any other patient or customer group in the market. In fact, this patients group could be considered as an experienced group because the therapy is very intense including weekly visits to therapists for many years [20].

### Data

#### Sample

The variables needed in econometric modelling were collected from several registers as well as carrying out questionnaires to the firms. During the contract period of 2007 to 2010, there were about 1,460 firms providing physiotherapy for disabled individuals and the amount decreased to 1,320 firms for the contract period of 2011 to 2014. In 2011 a total of 118 firms participated Kela’s service voucher pilot and approximately 1200 firms participated competitive bidding.

We used the DiD method in our effort to isolate the effect of the regulation, and, for purposes of this task, we have defined the regulated and unregulated firms and their quality before (the year 2007) and after the reform (the year 2011). We were able to gather data from 724 firms that had a contract with Kela during both periods. Our study group includes firms (n = 58) that were participating in competitive bidding in 2007 and were subject to price regulation in 2011. The control group (n = 666) includes firms that had determined their prices and quality in the tenders for both contract periods (2007-2010 and 2011-2014).

In order to control unobservable factors that could have an influence on the outcome, we have added other firm and market structure (municipality) level variables to the analyses. Our control variables are competition, potential patient capacity (describing firm size), average rental rate (a cost shifter) and the amount of disabled individuals receiving physiotherapy (a demand shifter). All of our control variables are justifiable and we face no bad control problem [21], because we are dealing with individual-level panel data and, e.g., competition (entry and exit of firms) is assessed not to be an outcome of the regulation^2^. By examining reimbursements before and after the regulation, Kela reimbursed physiotherapy (in the two pilot districts) for 112 firms in 2007 and for 105 firms in 2011. To avoid a possible bias regarding competition as a control variable, we also estimate the model without competition. In order to control possible changes in management styles etc. we also added firms types as dummy variables to the analyses^3^. For propensity score matching, we have also included the amount of population in a municipality, and firms’ risk rate (1-3) describing firms’ financial risk as additional controls to the estimation.

#### Data sources

Data regarding quality was collected after conducting questionnaires on the firms in 2013 and 2014 (fixed prices) or from Kela’s procurement department (market-determined prices). However, quality was scored by using the same scaling and hence quality is comparable despite different procurement and procuring mechanisms in 2007 and 2011. During competitive bidding, Kela evaluated and scored each tender’s price and quality information in a pre-defined manner. With service vouchers, on the other hand, only minimum quality requirements were verified by Kela, but excess quality of the service was not analysed or scored during the registration and, therefore, information on quality had to be gathered by conducting questionnaires on the firms. From a total of five questionnaires (and six reminders) that were sent to the firms in 2013 (January, February, March, April, November) – three of them were electronic and two of them were traditional post questionnaires. To gather more data, a total of 33 service providers were interviewed by phone in April 2014 [19].

Data regarding capacity and price were obtained from Kela, as was the data on the number of disabled individuals receiving physiotherapy in municipalities. The amount of population and the average level of rent in the municipalities were provided by Statistics Finland [22, 23], and information on the number of physiotherapists in the local market and firm level risk rates were obtained from Suomen Asiakastieto Oy.

Table 2 presents the average qualities and prices of regulated and unregulated firms. Table 3 provides descriptive statistics of the variables included in the estimation separately for the treatment and control groups.

**Table 2:** Mean quality and price of the regulated and unregulated firms

Year	Quality		Price	
	Regulated	Unregulated	Regulated	Unregulated
2007	73.34 (n = 58)	68.78 (n = 666)	41.79 (n = 58)	42.68 (n = 666)
2011	70.93 (n = 58)	82.67 (n = 666)	44.81 (n = 58)	47.81 (n = 666)

**Table 3:** Descriptive statistics

		Contract period 2011-2014 (post reform)				Contract period 2007-2010 (pre reform)			
	Variable details	Mean	S.E.	Min	Max	Mean	S.E.	Min	Max
Dependent variable			Treatment group/Control group				Treatment group/Control group		
Quality	Sum of quality factors scored during competitive bidding (control group) or with regulated prices (study group) scoring conducted by the researcher based on questionnaires (max score 103 points)	70.93/82.67	12.69/12.43	47/29	99/103				
Quality	Sum of quality factors scored during competitive bidding (max score 103 points)					73.34/68.78	12.07/10.82	48.03/27.20	96.66/100.66
Independent variables									
Competition (municipality)	Total number of physiotherapists (firms) per municipality	26.12/74.70	20.38/110.81	1/0	50/403	17.35/56.91	12.16/87.02	1/0	31/318
Capacity	Firm's potential patient capacity per year	30.98/34.05	34.30/41.71	2/0	220/330	22.35/18.92	27.86/20.45	1/5	150/200
Average rental rate (municipality)	Average rental rate (€/square meter) in a municipality. Average rental rate includes all rent realized in a privately financed market, not just rents of physiotherapists (firms).	8.85/10.07	0.84/2.09	8.00/7.87	10.00/14.92	8.08/8.80	0.92/1.50	7.12/7.12	9.33/12.01
Disabled individuals (municipality)	Total number of disabled individuals in a municipality receiving physiotherapy	122.45/234.56	90.26/272.09	9.00/7.87	227/963	110.81/205.27	85.44/244.27	11/3	233/856
Population (municipality)	Total population in a municipality	46147.53/112489.20	38370.42/164446.70	3436/1503	102308/595384	44710/108784	36888.14/156839.60	3564/1575	99308/568531
Risk rating	Firms are placed into different risk categories (1-3) based on their risk evaluation conducted by Suomen Asiakastieto Oy. Risk is calculated based on e.g. financial statement. Low risk =1, high risk =3	1.02/1.19	0.14/0.45	1/1	2/3	1.33/1.36	0.47/0.53	1/1	2/3

#### I. Dependent variable

The quality scoring was based on the same scoring as was conducted during competitive bidding organised by Kela in 2010 for the contract period 2011-2014 for physiotherapists not providing services with service vouchers. The quality scoring of price-regulated firms was carried by the researcher between January 2014 and April 2014. This ensured that the quality analysed was the same for both regulated and non-regulated firms. Firms that did not receive a contract with Kela were excluded. Our quality measure was previously used by Pekola et al. study - it is the sum of different quality factors and it could be described as the medium/long-term quality investments of a firm rather than as the quality of care [19]. Our quality measure includes education (max 20 points), work experience (max 30 points), the premises and their quality (max 6 points), the quality of the equipment (max 6 points) and the extent to which firms complied with Kela’s quality standards (max 41 points). The maximum quality score was 103 points. The qualities of the two contract periods were made comparable by multiplying the 2007 premises and their quality score points by 0.4, the equipment score by 1.2 and firms’ compliance with Kela’s quality standards by 1.17 because the original scoring differed between the two periods.

We argue that the quality parameters analysed in this study are valid because the scoring is uniform to all firms. Also according to the quality assurance standards by the Charted Society of Physiotherapy, quality physiotherapy includes a multitude of different quality factors [24] and Grimmer et al., for example, note that in addition to the outcome of care, the quality evaluation of physiotherapy may include different factors, such as the organisation of the service and the way in which care is provided [25].

#### II. Independent and control variables

We have added several firm and market structure level-independent variables into our analyses in order to control factors that could have an effect on the outcome. Our firm-level independent variables are: pre-reform quality (contract period 2007-2010), firms’ potential patient capacity for disabled individuals per year, which describes firm size and risk rating (1-3) based on, e.g., each firm’s financial statement. The market-level variables are: the number of competitors (firms providing physiotherapy) operating in the municipality, the average rental rate in a municipality [23], the number of disabled individuals receiving physiotherapy in a municipality, and the amount of population in a municipality [22]. For the final model, we also added a company-type dummy variable to the model in order to control for firm level time-invariant fixed effects. Firms producing physiotherapy are divided into six different company types. Self-employed therapists are the largest group (approximately 40%). Other company types are limited partnership, partnership, limited company, foundation and association.

## Methods

When analysing the effects of regulation, one approach is to compare regulated and unregulated firms or markets [26]. Stigler and Friedland’s paper on regulated electricity prices is a good example of this method of analysis [27]. We used the DiD method in our effort to isolate the effect of price regulation, and for this task we have defined the regulated and non-regulated firms and their quality before and after the reform. The coefficient of interest (the interaction term) forms after the average gain over time in the control group is subtracted from the average gain over time in the treatment group. The method basically removes biases that could either be caused by permanent differences between the two groups or biases resulting from time trends unrelated to the regulation [28].

The basic model (model 1) of interest is the following

1 $$ {y}_{i t}=\alpha +\beta {D}_i+\gamma {T}_t+\theta {D}_i\times {T}_t+{W}_{i t}\tau +{\varepsilon}_{i t} $$

where y is the outcome of interest e.g. quality, α is the constant term, *D*_*i*_ is a dummy variable identifying (firms’) treatment and control groups (D_i_ = 1 study group, D_i_ = 0 control group) to be called Price regulation, *T*_*t*_ is the time indicator (T_t_ = 0, if t = 2007, and T_t_ = 1, if t = 2011) to be called Time, and D_i_ × T_t_ is the main interaction variable (hereafter called quality effect) of the DiD-estimation, and ε_it_ is the error term. The control variables used in the estimation were included in the vector W_it_.

Despite DiD regression is a fairly precise mechanism for estimating the effect of a treatment or a reform with non-experimental data, there are certain well-known caveats with the DiD analyses. Parallel trend assumption is one of the most common problems with DiD estimation and, therefore; it should be tested that the two groups did not differ before the reform was implemented. Unfortunately we did not have access to additional data regarding (multiple) periods prior and post reform to have a better understanding of the parallel trend assumption in the quality of physiotherapy. However, in spite of this we use individual-level panel data, which enables us to control factors that vary across firms and factors that are unobservable. We also aim to control factors that could have an effect on quality for other reasons than price regulation and, therefore, for the model 2 we have added previously mentioned pre-reform and time-varying control variables to increase the precision of our estimates.

Another robustness check is executed with a slightly different quality measure. As mentioned earlier, our original quality measure is the sum of different quality factors that were scored either during the procurement process (competitive bidding) or after firms replied to questionnaires that were sent during the research. One of the quality factors (firms’ compliance with Kela’s quality standards) was difficult to score outside the procurement process, because the scoring involves judgement and, therefore, for the model 4, we modified our quality variable by removing this particular quality indicator. Finally, we also added firm type dummy variables to the model in order to control for firm type time invariant factors in our analyses.

As there are previously mentioned deficits in DiD estimation and our data, in the final stage, we tested the robustness of our DiD estimates as well as the unobservable group-specific pre-regulation heterogeneity between the study group and the control group with Kernel Matching (KM) and balancing properties respectively. The basic idea with propensity score matching is to find matches for treated units from the control group [29]. Kernel matching uses all treated units and all controls in its estimation and thus this matching algorithm is used in this study because the number of treated firms is fairly small. To increase the precision of the matching we also bootstrapped standard errors. Based on Rosenbaum and Rubin, matching is a method of selecting units from the control group that are similar to units in the study group with respect to the distribution of observed covariates [30, 31]. The balancing test on the other hand, performs a balancing *t*-test of difference in means of the specified covariates between the control and treated groups during the pre-regulation period [32].

## Results

Kela piloted regulated price service vouchers in two insurance districts during the contract period 2011-2014. Firms located in these districts had fixed prices and the prices needed to cover all costs of the service, as firms were not allowed to charge any extra fees from patients. On the other hand, patient choice was also initiated in 2011 for the same service. Free choice was granted to all patients despite the procurement mechanism.

Based on the previously described system, the service voucher reform could have induced firms to change their behaviour regarding quality. Based on theory regarding price regulation and quality competition, the effect on quality due to the reform is unambiguous. We have tested by using empirical data from physiotherapy, which theoretical prediction dominates the market.

We used DiD estimation techniques as well as Kernell matching in our effort to isolate the effect. We used firm level pre- and post-regulation data in our estimations. The data included both regulated and unregulated firms. We also used several control variables in the estimations.

Our results from the first model (Table 4) indicate that the quality of firms participating in service voucher pilots had decreased, but the study group and the control group differed from each other statistically significantly (at the 5% level) and thus the results could have been biased.

**Table 4:** Full results from DiD regression for quality (model 1)

Model		Coefficient	Standard error	P > |t|
Quality				
Time		13,8936	0,64	***
Price regulation		4,5663	1,60	**
Quality effect		−16,3050	2,27	***
Constant		68,7760	0,45	***
Significance level: 0,05% = *, 0,01% = **, =0.001 = ***				
N	1448			
F (3,1444)	160,00			
Prob > F	0,0000			
R-squared	0,2495			
Adj R-squared	0,2479			
Root MSE	11,71			

By adding control variables to the model, we aimed to control unit-specific changes between the periods and the results from model 2 (Table 5) show that the reform indeed had a negative and statistically significant effect on quality, and the use of the control variables diminished the difference between the two groups as the difference was no longer statistically significant. By removing the competition variable from the models, we aimed to remove the possibility of bad controls. However, the removal did not alter the results.

**Table 5:** Full results from DiD regression for quality (model 2)

Model		Coefficient	Standard error	P > |t|
Quality				
Time		12,9472	1,11	***
Price regulation		0,3139	2,77	
Quality effect		−14,7613	3,10	***
Competition		−0,0072	0,02	*
Capacity		0,0537	0,01	***
Rent		0,4706	0,42	
Disabled individuals		0,0010	0,01	**
Quality 2007		0,7039	0,03	***
Constant		15,0494	2,91	***
Significance level: 0,05% = *, 0,01% = **, =0.001 = ***				
N	1065			
F (8,1056)	205,82			
Prob > F	0,0000			
R-squared	0,6093			
Adj R-squared	0,6063			
Root MSE	8,6152			

Our third model includes a modified quality measure because one aspect of the quality (firms’ compliance with Kela’s quality standards) was difficult to score outside of the procurement process and this difficulty could have caused problems in measuring the outcome. We continued to analyse only those firms that had a contract during both periods and results from the modified quality measure (Table 6) support our previous findings that the reform had a negative and statistically significant effect on quality. However, the effect is much more modest in this model. This could mean two things: either the difficulty of the scoring indeed overestimated the results (a negative effect) or firms decreased their quality most in this respect due to price regulation. The firms’ compliance with Kela’s quality standards is probably the easiest quality factor to decrease because other quality factors (such as a firm’s equipment or its premises) are likely to react more slowly to price regulation. Finally, we also added company-type dummy variables to our regression to control for firm level time-invariant factors, but the main result was not altered (Table 7).

**Table 6:** Full results from DiD regression for modified quality variable (model 3)

Model		Coefficient	Standard error	P > |t|
Quality				
Time		14,0588	0,51	***
Price regulation		−0,7175	1,43	
Quality effect		−6,7281	1,71	***
Competition		0,0029	0,01	
Capacity		−0,0030	0,01	***
Rent		0,2264	0,23	
Disabled individuals		−0,0030	0,00	
Quality 2007		0,5442	0,02	***
Constant		−8,1035	2,34	**
Significance level: 0,05% = *, 0,01% = **, =0.001 = ***				
N	1064			
F (9,1055)	243,35			
Prob > F	0,0000			
R-squared	0,6485			
Adj R-squared	0,6459			
Root MSE	6,9245

**Table 7:** Full results from DiD regression with company type dummy variables

Model		Coefficient	Standard error	P > |t|
Quality				
Time		14,1022	0,51	***
Price regulation		−0,9338	1,43	
Quality effect		−6,6596	1,71	***
Competition		0,0007	0,01	
Capacity		0,0281	0,01	***
Rent		0,2706	0,23	
Disabled individuals		−0,0024	0,00	
Quality 2007		0,5524	0,02	***
Company type 1^a^		−2,6226	1,81	
Company type 2		−2,9991	1,53	
Company type 3		−2,3737	1,48	
Company type 4		−3,4304	1,87	
Company type 5		−3,7229	1,47	*
Constant		−5,7691	2,75	***
Significance level: 0,05% = *, 0,01% = **, =0.001 = ***				
^a^ = foundation treated as a reference group				
N	1064			
F (13,1050)	151,39			
Prob > F	0,0000			
R-squared	0,6521			
Adj R-squared	0,6478			
Root MSE	6,9057			

Lastly, as a robustness check, we completed a kernel matching and, as background analysis for this, we first estimated the probability of a firm’s participation in price regulation with the probit regression. Results from the probit regression (Table 8) indicate that price-regulated firms are likely to have less competition in their area (market) but there are also more disabled residents receiving physiotherapy. The firms also have slightly higher quality before the reform, have somewhat lower financial risk and have more staff per firm (yet weakly). The results regarding, e.g., competition are sensible, as price regulation was implemented in medium-sized insurance districts.

**Table 8:** Results from the probit regression

Model		Coefficient	Standard error	P > |t|
Pricereg				
Competition		−0,0348	0,0084	***
Disabled individuals		0,0060	0,0021	**
Population		0,0000	0,0000	
Risk rating		−0,3944	0,1574	*
Rent		−0,1420	0,0725	
Staff		0,0034	0,0015	*
Quality_2007		0,0124	0,0054	*
Constant		−0,5608	0,7438	
Significance level: 0,05% = *, 0,01% = **, =0.001 = ***				
N	1054			
LR chi2 (6)	74,01			
Prob > chi2	0,0000			
Pseudo R2	0,1185			

**Fig. 2:**
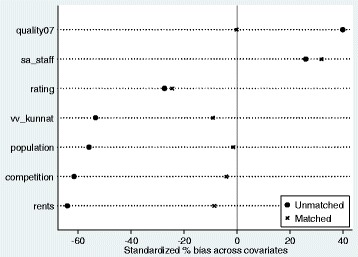
Balancing of the properties between treatment and control groups

As is suggested by previous literature [33], outcome values for quality after the reform are not included in the matching process. The balancing properties were also satisfied by using a set of baseline covariates. We were able to find 567 matches from the control group for 92 regulated firms. The description of the estimated propensity scores in regions of common support are presented in Table 9. Overlap between treatment and comparison groups is presented in Fig. 2. As can be seen, the density distribution of the propensity score is satisfying after the matching.

**Table 9:** Description of the estimated propensity score in region of common support

Model	Percentiles	Smallest
1%	0,0538	0,0529
5%	0,0586	0,0530
10%	0,0640	0,0531
25%	0,0815	0,0532
50%	0,1103	
Largest		
75%	0,1526	0,4220
90%	0,1998	0,4494
95%	0,2472	0,5516
99%	0,3824	0,9894
The region of common support is [0.0529, 0.9894]		
Obs	656	
Mean	0,1276	
Stand. Dev.	0,0733	
Variance	0,0054	
Skewness	3,9020	
Kurtosis	34,9751

Conversely, the results from the matching (Table 10) confirm our findings that the reform indeed had a negative and statistically significant effect on quality. The result of the average treatment effect of the treated is uniform (approximately –6 quality points) with the DiD analyses with control variables and a modified quality variable (Table 6). These additional identical results confirm that our results are unbiased and the negative effect on quality was caused by the service voucher reforms.

**Table 10:** Results from the kernel matching with bootsrapped standard errors

Variable	Reps	Observed	Bias	S.E.	95% conf. Intervall	
Quality	100	−5,7311	0,049582	1,439388	−8.5872-2.8750	Normal
					−8.7314-2.3824	Percentile
					−8.7316-2.3824	Bias-corrected

**Fig. 3:**
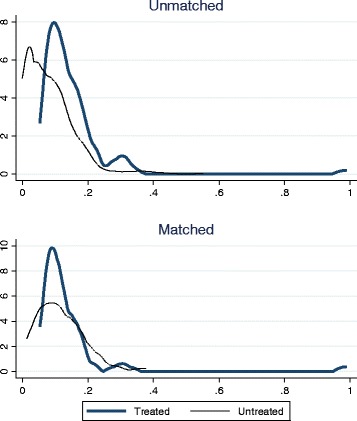
Balancing of the properties between treatment and control groups

**Fig. 4:**
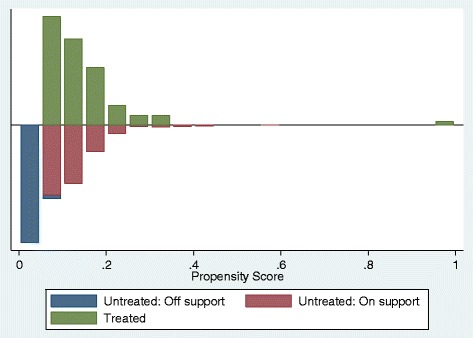
Common support of regulated and non-regulated firms

Balancing properties as well as common support of regulated and non-regulated firms are presented in Figs. 3 and 4. On a final note, it is important to mention that the balancing of the covariates describing the firms (Table 11) before the regulation is satisfactory and thus we conclude that our results are robust.

**Table 11:** Results from the balancing of covariates describing the firms included in the study

Variable	Unmatched	Mean				*t*-test			
	Matched	Regulated	Non-regulated	%bias	reduct % bias	|t|	p > |t|	V (T)/V (C)	
Competition	U	21,7330	65,828	−61,4		−4,74	***	0.03^a^	
	M	25,3920	17,706	10,7	82,6	1,66		0,59	
Disabled individuals (municipality)	U	122,4500	234,24	−53,4		−4,27	***	0.11^a^	
	M	122,4500	140,16	14,5	72,9	1,39		0,62	
Population	U	46148	1,10E + 05	−55,9		−4,36	***	0.05^a^	
	M	46148	47888	11,1	80,2	1,6		0,70	
Number of staff	U	36,7270	8,361	25,9		4,93	***	18.63^a^	
	M	36,7270	4,9444	26,6	−2,4	1,09		17.12^a^	
Average rental rate (municipality)	U	8,8493	10,065	−64,0		−5,39	***	0.25^a^	
	M	8,8493	8,9803	15,3	76,1	1,27		0,62	
Risk rating	U	1,0189	1,1913	−27,3		−2,30	*	0.55^a^	
	M	1,0189	1,125	4,5	83,5	1,00		-^a^	
Quality_2007	U	73,3420	68,786	40,0		4,32	***	1,23	
	M	73,3420	73,36	14,2	64,6	0,72		1,45	
^a^ if variance ratio outside [0.69; 1.44] for U and [0.57; 1.75] for M									
Results are called by using psmatch2 in Stata									
		Ps R2	LR chi2	p > chi2	MeanBias	MedBias	B	R	% Var
Sample									
Unmatched		0,119	74,01	0,000	46,9	53,4	75.1^b^	0.09^b^	86
Matched		0,039	5,46	0,486	13,8	14,2	46.1^b^	0,66	29
^b^ if B > 25%, R outside [0.5; 2]									

A regulatory policy that sets a price as a markup above marginal costs yields to the socially optimal price and quality if the regulator had full information about the market and its behavior [34]. If the regulated price is set under marginal costs, firms have an incentive to diminish quality. Finally, by using t-tests, we tested the differences between the average regulated prices and the prices determined by firms in the tenders before the price regulation was implemented (Table 12).

**Table 12:** Results from t-tests regarding prices before and after the regulation

	obs	mean	se	sd	95% conf. Interval	
Pre regulation price	96	43,36	0,63	6,22	42,10	44,62
Post regulation price	118	43,89	0,34	3,70	43,21	44,57
Combined		43,65	0,34	4,99	42,98	44,32
Diff		−0,53	0,69		−1,89	0,82
t = -0.7733						
degrees of freedom = 211						
Ha:diff < 0	Ha:diff = 0		Ha:diff > 0			
Pr (T < t) = 0.2201	Pr|T| > |t| = 0.4402		Pr (T > t) = 0.7799			

The regulated price was negligibly different statistically from the pre-regulation period prices when the earnings index was controlled. Therefore it could be stated that the quality decrease was not caused by an inappropriate level of regulated price. Most likely, our results are caused by firm’s efforts to cut costs due to price regulation but it seems that this cost reduction was made by decreasing quality. We argue that competition did not incentivise firms to compete for patients on quality. It is likely that unresponsiveness of firms to quality competition is caused by imperfect information. Despite free choice having been initiated in 2011, comparable quality information is not provided for patients that would support their decision making. Therefore, the results from the empirical estimations are sensible and support theoretical findings that price regulation tends to decrease quality in health care.

## Discussion

With this study, we are able to analyse price regulation combined with the free choice of patients (i.e., competition) in physiotherapy. Our findings show that quality was decreased due to the reform. Similar reform is planned for public health care in Finland and this is the first study that uses Finnish data in analysing firms’ incentives towards price regulation and free choice of patients. The aim was to test which theoretical prediction dominated in the market – price regulation and the possibility to cut costs through quality or quality competition, which by the general theory of competition with fixed prices should enhance quality unless factors such as imperfect information influence the incentives of firms.

Based on our findings, quality reduction was statistically significant in all models. All of our regression models, as well as KM, show that quality was reduced due to the service voucher reform which had fixed prices but also introduced free choice of patients. Most likely the result is caused by price regulation. Fixed prices alter the financial incentives of firms and Ellis has shown that patient selection and quality discrimination of hospitals is sometimes even boosted under competitive environment [4]. Also Meltzer et al. [3] present in their study that an increasingly competitive environment under fixed prices has the effect of increasing quality the most for the least costly patients. Gravelle and Masiero on the other hand point out the incentive effects of providers are lower in any capitation fee when information is imperfect [17].

The more a health care provider, e.g., a hospital provides services under fixed prices, the lower the net revenue it receives. Thus, the success of the pricing must be evaluated through the interests of patients and providers [35]. Due to the imperfect information, the interest of patients is difficult to stand out despite free choice and thus the financial incentives of firms regarding price regulation is solved by reductions in quality. The result is sensible as the evaluated quality marks quality investments of firms rather that the outcome of care.

Even though our results seem solid, ideally the assessment of the regulation to the behavior and performance of firms requires a fairly lengthy time series to avoid basing conclusions on possible transitional responses [26]. Unfortunately, we did not have access to several pre- or post-treatment periods and we were unable to test, e.g., pre-treatment trends of the regulated and un-regulated groups with alternative parallel assumptions as suggested by Angrist & Pischke [21]. This is definitely a shortcoming of our study. Conversely, there are several issues that support the fact that firms were alike in both study and control groups. Firstly, all firms had to participate in competitive bidding before the implementation of the fixed-price service vouchers. Secondly, all firms had similar contracts with Kela and all firms were treating disabled individuals (criteria of the disabled were defined by Kela). Finally, all firms had to follow the minimum quality criteria defined by Kela. These issues support our understanding that the firms in both groups were similar before the reform.

Another weakness of the DiD regression lies in the unobserved temporary effect (e.g., change of study groups’ behavior prior to the implementation of the price regulation) also known as Ashenfelter’s dip. In our study, this means that the quality of the firms needed to decline before the price regulation, which conversely, would have overestimated the impact of price reform and biased our estimate. However, pre-regulation quality decline is not a possible option in our study because prior to the price regulation, all firms had to take part in competitive bidding and had contracts with Kela, which strongly forbids quality decline during the contract period and controls it with different contractual penalty instruments. For this reason, the negative change in quality had to happen after the price regulation was implemented and was not an anticipation effect. An additionally compositional effect over time should not cause problems in this study as both regulated and unregulated firms are not going to get mixed. In our study, there is no such case in which firms with regulated insurance districts would have a chance to influence the prices or participate in competitive bidding instead and therefore, before and after, comparability is not compromised.

Unfortunately due to missing data we were unable to perform proper response bias analyses on firms which participated in competitive bidding and had non-regulated prices during both periods but were not included in the study. However, firms which participated in a service voucher pilot (and had regulated prices) and were not included in the data of this study (due to missing quality data from both periods) have been previously analysed by Pekola et al. [20]. The results indicate that firms that had regulated prices but were not included in this study were smaller firms (based on potential patient capacity) and perhaps had lower than average quality.

## Conclusion

Our study shows that quality was decreased due to the reform which regulated prices but also initiated free choice of patients. We aimed to analyse which of the two mechanisms dominated in the market - cost containment due to price regulation or quality competition due to free choice of patients. As all of our regression models as well as our sensitivity analyses using kernel matching present similar results, we conclude that our results are robust. However, due to the growing interest of using price regulation and competition in health care and the fact that price regulation is sometimes associated with decreased service quality it is important to discuss means aimed to target firms’ unwanted behavior towards quality.

Regulators have invented different mechanisms, such as revenue-share penalties (used in different industries such as telecommunications and electric power), which are designed to eliminate this undesirable behavior, but paradoxically, they may in fact encourage firms to do just the opposite - reduce investments in quality [36]. On the other hand, even Arrow mentions in his famous 1963 paper regarding the physician market that risk and uncertainty are significant elements of health and, therefore, information ends up having a market on its own [37]. As quality information of the service provided by the physicians is not apparent upon inspection by patients, quality deteriorates to the lowest level in the market, causing serious market failure [38].

However, by increasing information regarding the service quality of firms and initiating benchmarking has been shown to increase investments in quality [36]. We strongly believe that actions aiming to increase patients’ knowledge about service providers are needed in physiotherapy. Also benchmarking could have a positive effect on quality as well. Ultimately, it is undisputed that when patient choice is more and more widely introduced, different mechanisms that enhance information must be developed in order to enhance the ability of patients to choose providers, but also to incentivise providers toward quality investments. This also presumably has an impact on the financial incentives of firms in their effort to cut costs through quality reductions when prices are fixed.

## Abbreviations

DiD: Difference-in-Differences
DRG: Diagnosis-Related Group
GP: General Practitioner
IRF: Inpatient rehabilitation facilities
Kela: The Social Insurance Institution
KM: Kernel Matching
PPS: Prospective payment system

## Footnotes

1 Minimum quality criteria for firms participating in a service voucher pilot is fairly close to the minimum quality criteria for firms participating in competitive bidding. Minimum criteria linked with service voucher pilot include several issues, e.g., the firm must be entered into the prepayment register, insurance for their business (patient insurance and liability insurance) are required, their premises and equipment must be safe for disabled individuals, they must accept regulated prices and declare that they would not charge the patients any extra fees, they need to inform Kela of any changes to their business, therapists must have professional practice rights, they must have adequate first aid skills and they must be acquainted with Kela’s quality standard.

2 Due to the missing data a precise figure of firms entering and exiting the market studied may not be provided. However, based on the existing data approximately 22% of the firms exited the market after the period 2007-2010. In contrast, approximately 16% of the firms entered the market as new providers in 2011 and the figures were similar between regulated and non-regulated areas.

3 Approximately 48% of firms studied are self-employed, 26% are limited companies, 16% are limited partnerships and the rest (10%) are general partnerships, foundations and associations.
